# Fecal bile acid excretion and messenger RNA expression levels of ileal transporters in high risk gallstone patients

**DOI:** 10.1186/1476-511X-8-53

**Published:** 2009-12-08

**Authors:** Jorge Herrera, Ludwig Amigo, Constanze Husche, Carlos Benítez, Silvana Zanlungo, Dieter Lütjohann, Juan Francisco Miquel, Flavio Nervi

**Affiliations:** 1 Departamento de Gastroenterología, Facultad de Medicina, Pontificia Universidad Católica de Chile, Santiago, Chile; 2 Labor für spezielle Lipiddiagnostik, Zentrum Innere Medizin, Institut für Klinische Chemie und Pharmakologie, Universitätsklinikum Bonn, Germany

## Abstract

**Background:**

Cholesterol gallstone disease (GS) is highly prevalent among Hispanics and American Indians. In GS, the pool of bile acids (BA) is decreased, suggesting that BA absorption is impaired. In Caucasian GS patients, mRNA levels for ileal BA transporters are decreased. We aimed to determine fecal BA excretion rates, mRNA levels for ileal BA transporter genes and of regulatory genes of BA synthesis in Hispanic GS patients.

**Results:**

Excretion of fecal BA was measured in seven GS females and in ten GS-free individuals, all with a body mass index < 29. Participants ingested the stool marker Cr_2_O_3 _(300 mg/day) for 10 days, and fecal specimens were collected on the last 3 days. Chromium was measured by a colorimetric method, and BA was quantitated by gas chromatography/mass spectroscopy. Intake of calories, nutrients, fiber and cholesterol were similar in the GS and GS-free subjects. Mean BA excretion levels were 520 ± 80 mg/day for the GS-free group, and 461 ± 105 mg/day for the GS group. Messenger RNA expression levels were determined by RT-PCR on biopsy samples obtained from ileum during diagnostic colonoscopy (14 GS-free controls and 16 GS patients) and from liver during surgery performed at 8 and 10 AM (12 GS and 10 GS-free patients operated on for gastrointestinal malignancies), all with a body mass index < 29. Messenger RNA level of the BA transporter genes for ileal lipid binding protein, multidrug resistance-associated protein 3, organic solute transporter alpha, and organic solute transporter beta were similar in GS and GS-free subjects. Messenger RNA level of Cyp27A1, encoding the enzyme 27α-hydroxylase, the short heterodimer partner and farnesoid X receptor remained unchanged, whereas the mRNA level of Cyp7A1, the rate limiting step of BA synthesis, was increased more than 400% (p < 0.01) in the liver of GS compared to GS-free subjects.

**Conclusion:**

Hispanics with GS have fecal BA excretion rates and mRNA levels of genes for ileal BA transporters that are similar to GS-free subjects. However, mRNA expression levels of *Cyp7A1 *are increased in GS, indicating that regulation of BA synthesis is abnormal in Hispanics with GS.

## Background

Cholesterol gallstone disease (GS) is prevalent worldwide, and is endemic among American Indians and Hispanics [1,2]. In GS, the primary abnormality is secretion by the liver into the gallbladder of bile that is supersaturated with cholesterol. This leads to precipitation of cholesterol and formation of gallstones. The most common causes of cholesterol-saturated bile are a decrease in the bile acid (BA) pool and secretion rate and an increase in cholesterol output into the bile [3,4]. Thus, one current hypothesis about the primary cause of GS is abnormal regulation of cholesterol and BA metabolism.

Fecal BA excretion rate is a marker of daily BA synthesis, and is higher in Argentinean Hispanic patients with GS than in GS-free individuals [5]. Similarly, BA synthesis, indirectly assessed by the measurement of serum 7α-hydroxy-4-cholesten-3-one concentration in one point in time, was found increased in Chilean Hispanics with GS compared to GS-free individuals, suggesting increase BA synthesis in GS [6,7]. Similar results were observed in Caucasian individuals [8,9]. Consistent with these findings, the mRNA levels of BA transporter genes are decreased in the ileum of female Caucasian GS patients, supporting the hypothesis that absorption of BA is impaired and fecal BA excretion is increased in subsets of GS patients [10,11]. GS-free North American Indians, a population with an extremely high prevalence of GS, also have higher fecal BA excretion rates than non-Indian subjects [12]. However, GS patients who are North American Indians [12] or obese non-Indians [13] have fecal BA excretion levels that are similar compared to levels found in GS-free subjects. Consistent with these findings, other studies performed among Italian [14] and Chinese [15] subjects have shown unchanged mRNA expression levels of CYP7A1, the ratelimiting enzyme of BA synthesis.

These apparent discrepancies might be because the mechanisms of GS formation are different in patients with different ethnic backgrounds, or because indirect assessment of BA synthesis in one part of the day might not reflect total daily production of BA, as occurs with the measurement of daily fecal BA excretion. Thus, the objective of this study was to quantitate daily fecal BA excretion rates, mRNA expression levels of ileal BA transporters and of hepatic regulatory genes of BA synthesis, in GS patients from a high-risk Hispanic population.

## Results

Nutrients and caloric intake were similar in GS-free control subjects and GS patients in this study of fecal BA excretion (results not shown). Caloric intake varied from 1220 to 2237 Kcals/day for all subjects. Fiber intake varied between 19.3 and 24.1 g/day, and cholesterol intake varied between 94.4 and 281.3 mg/day, and both were similar in GS-free and GS patients. All subjects were non-obese and had similar values for age, body mass index (BMI) and serum lipids (Table 1). No significant differences were observed in daily fecal BA excretion rates between the GS-free and GS patient groups, with rates varying from 186 ± 53 (mean ± standard error) and 883 ± 53 mg/day in the GS-free patients, and from 120 ± 17 and 897 ± 157 mg/day in the GS group (Table 1).

**Table 1:** Age, BMI, serum lipids and fecal BA excretion in control and GS patients.

			Serum lipid concentration (mg/dl)			
Subjects	Age (y)	BMI	Total CH	HDL	TG	Fecal BA (mg/day)
**GS-free**						
1	48	23.4	170	52	99	883 ± 53
2	43	23.4	223	46	73	824 ± 68
3	46	22.2	219	59	220	418 ± 140
4	36	23.7	219	67	57	880 ± 150
5	22	28.3	194	58	81	250 ± 54
6	48	22.1	226	76	68	428 ± 140
7	34	22.4	153	60	38	420 ± 127
8	36	26.3	192	50	100	403 ± 140
9	37	24.1	158	37	111	186 ± 30
10	55	23.0	247	66	104	508 ± 250

**Mean ± SE**	**41 ± 3**	**24.1 ± 0.6**	**198 ± 10**	**57 ± 4**	**95 ± 16**	**520 ± 80**

**With gallstones**						
11	55	23.8	247	66	104	374 ± 9
12	51	22.5	163	50	65	576 ± 250
13	30	22.5	150	39	165	314 ± 86
**Cholecystectomy**						
14	47	22.1	150	28	177	324 ± 46
15	53	22.0	148	50	118	120 ± 17
16	37	24.1	162	44	146	897 ± 157
17	30	26.7	150	39	165	538 ± 170

**Total GS**						
**Mean ± SE**	**47 ± 4**	**23.4 ± 0.6**	158 ± **3**	**46 ± 4**	**114 ± 19**	**461 ± 105**

Each individual value of fecal BA excretion represents the mean ± SE of the three days of fecal collection of each participant. Body weights were: GS-free subjects, 58.6 ± 1.9; GS patients, 55.3 ± 1.7. BMI was calculated as weight/height (kg/m^2^); CH, cholesterol; TG, triglycerides.

The mRNA levels of genes involved in the regulation of the enterohepatic circulation of BA in the ileum were analyzed in GS and GS-free subjects with similar average ages and BMIs. Histological analysis of ileal biopsies showed no abnormalities for either GS-free or GS patients. As shown in Table 2, no differences were observed between GS patients and GS-free subjects in the mRNA levels of BA transporter genes, including the apical sodium dependent bile acid transporter (*Astb*); *Fabp6*, which is the gene for ileal lipid binding protein (ILBP); *Abcc3*, which is the gene for multidrug resistance-associated protein 3 (MRP3); or *Ostα and Ostβ*, which are the genes for the organic solute transporters alpha and beta. Similarly, no changes were seen in the expression levels of FGF19, the ileal hormone that regulates BA synthesis.

**Table 2:** Expression levels of mRNA of ileal transporters of BA and FGF19 in GS patients.

Gene symbol	Gene ID	Expression value in GS	P value
*Slc10A2*	6555	0.99 ± 0.19	0.964
*Fabp6*	2172	1.01 ± 0.11	0.983
*Abcc3*	8714	1.08 ± 0.07	0.586
*Ostα*	200931	1.18 ± 0.11	0.425
*Ostβ*	123264	1.02 ± 0.08	0.902
*Fgf19*	9965	1.30 ± 0.20	0.411

Expression represent the mean values ± SE of GS subjects. Expression values for GS-free subjects were assigned as 1.00. Participants of the study were: GS, n = 16 (16 GS, 12F/4 M and 7), GS-free subjects, n = 14 (8 F/6 M). Age ranges were 62 ± 2 for GS-free, 57 ± 3 for GS. Gene ID corresponds to gene annotation obtained from NCBI web site. Expression of the following genes were determined: *Slc10A2*, ASBT (Apical sodium dependent bile acid transporter); *Fabp6*, ILBP (Ileal Lipid Binding protein); *Abcc3*, MRP3 (Multidrug resistance-associated protein 3); *Ostα*, Organic solute transporter alpha; *Ostβ*, Organic solute transporter beta; *Fgf19*, Fibroblast growth factor 19.

The mRNA levels of regulatory genes of BA synthesis were determined in liver biopsies obtained from a series of GS and GS-free subjects subjected to laparotomy for GS, or for localized gastrointestinal malignancies, respectively. Histological analysis of liver biopsies was normal for either GS-free or GS patients. As shown in Table 3, the mRNA level of Cyp7A1, the gene encoding 7α-hydroxylase, the rate limiting step of BA synthesis, was increased by >400% (p < 0.01) in GS compared to GS-free patients. No differences were observed between GS patients and GS-free subjects in the mRNA levels of Cyp27A1 and of regulatory genes of BA synthesis, including *Nr0B2 *(short heterodimer partner) and *Nr1H4 *(farnesoid X receptor, FXR).

**Table 3:** mRNA expression levels of hepatic regulatory genes of BA synthesis in GS patients.

Gene symbol	Gene ID	Expression value ± SE	p value
*Cyp7A1*	1581	4.12 ± 2.68	0.012*
*Cyp27A1*	1593	1.10 ± 0.45	0.717
*Nr0B2*	604630	1.08 ± 0.52	0.788
*Nr1H4*	603826	0.83 ± 0.28	0.523

Expression values represent the mean ± SE of GS subjects. Expression values for GS-free subjects were assigned as 1.00. Participants of the study were: GS, n = 12 (9 F/3 M), GS-free subjects, n = 10 (5 F/5 M). Age ranges were 50 ± 4 for GS and 56 ± 5 for GS-free. Gene ID corresponds to gene annotation obtained from NCBI. *Cyp7A1*, cytochrome P450, family 7, subfamily A, polypeptide 1 (cholesterol 7α-hydroxylase); *Cyp27A1*, cytochrome P450, family 27, subfamily A, polypeptide 1 (sterol 27α-hydroxylase); *Nr0B2 *nuclear receptor subfamily 0, group B, member 2 (short heterodimer partner); *Nr1H4*, Nuclear receptor subfamily 0, group H, member 4 (farnesoid x receptor, FXR).

## Discussion

This study found similar rates of daily BA excretion in the feces of Chilean Hispanic GS patients and GS-free individuals. Our observations do not support the results of Mamianetti et al., who found increased BA excretion levels in the feces of GS patients compared to GS-free subjects [5]. However, since that study was performed without a fecal marker to correct for fecal flow, its findings are difficult to interpret, and cannot be compared to our study and other similar studies in which fecal flow markers were used to assess sterol excretion and balance [16-18]. Other studies that have reported fecal BA excretion rates in GS patients show similar daily rates of BA excretion in the feces of GS and GS-free subjects [12,13].

Consistent with the apparently normal daily fecal BA excretion in GS patients, we found that GS-free and GS patients had similar mRNA levels for BA transporter genes in the ileum. This observation suggested that this group of GS patients had no abnormalities in ileal BA absorption, in contrast with the decreased mRNA levels of BA transporters observed in the ileum of Caucasian female GS patients in other studies [10,11]. Our results indicate that Chilean Hispanics with GS maintain a normal rate of BA absorption in the ileum. However, a specific abnormality in the expression levels of ileal BA transporters in GS patients may not be detected in this population because of the extremely high prevalence of GS among Chileans, who were used as both patients and controls in this study.

The finding that fecal BA excretion is unchanged in our GS patients is consistent with normal rates of daily BA synthesis determined by isotopic methods in GS [19,20] and with normal mRNA expression levels of CYP7A1 found among Italian [14] and Chinese [15] GS patients. These observations apparently contradicts the finding of increased mRNA levels of CYP7A1 of the present study and the previously reported increase in 7α-hydroxy-4-cholesten-3-one in serum in Hispanics [6,7], as well as in Caucasian GS patients [8,9]. The most likely explanation for this apparent contradiction is that the increased CYP7A1 mRNA and serum 7α-hydroxy-4-cholesten-3-one concentration seen in GS patients was observed at a single time point, measured by an indirect assessment of BA synthesis. Thus, the values may not reflect the daily rate of BA synthesis as measured by the daily fecal BA excretion method, or by isotopic methods. BA synthesis shows a rapidly changing circadian rhythm in rodents [21] and in humans [22]. Therefore, the increase in BA synthesis, assessed by either CYP7A1 mRNA, or a serum marker of BA synthesis measured at only one point during the day, may indicate a secondary phenomenon that is an effect of the loss of gallbladder function in some populations, and not a primary pathogenic defect of all GS patients. Whether the mechanism responsible for the increase mRNA expression levels of CYP7A1 in GS is mediated by the FXR nuclear receptor - dependent or FGF 19 - dependent mechanism, cannot be elucidated with the results obtained in these studies. It is possible that the fraction of the BA pool stored in the intestine during the night is higher in GS patients compared to GS-free subjects. This situation could decrease BA concentration in hepatocytes and consequently, decrease FXR activity followed by increase of mRNA levels of CYP7A1 early in morning, when liver biopsies and serum samples are obtained from the subjects participating in these studies. Alternatively, loss of gallbladder function because of the presence of gallstones, or cholecystectomy could change the daily secretion pattern of FGF19 with the subsequent modification of the circadian rhythm of CYP7A1 mRNA.

The fecal BA excretion rates previously reported for North American Indians are higher than rates observed for Caucasian individuals [12,16-18], but similar to the values reported here. This suggests that Amerindians may have higher rates of fecal BA excretion than other populations, which might represent a common ethnic predisposition to form gallstones, resulting from a reduced capacity for BA absorption in the ileum. Another possibility relates to the presence of common dietary constituents in the diet of Amerindians and Hispanics that might increase fecal BA excretion and cholesterol saturation in bile such as occurs with legume diets [18,23], a common foodstuff in these populations [23,24]. Specific types of fiber, such as the saponin component in the soluble fraction of legumes, may increase the synthesis of BA [25,26] and favour its excretion in feces [18]. Interestingly, the levels of fecal BA excretion reported here in GS patients and GS-free subjects were similar to values reported for Caucasian subjects ingesting a diet high in fiber [16] or in legumes [18].

## Conclusion

Fecal BA excretion, a marker of daily BA synthesis, and mRNA expression levels of ileal transporters remain unchanged in GS compared to GS-free Hispanic subjects. Increased CYP7A1 mRNA expression levels found in GS compared to GS-free subjects, suggests that regulation of BA synthesis is abnormal in GS patients from Hispanic populations.

## Methods

### Patient selection and sample collection

The Institutional Review Board for Human Studies of the Faculty of Medicine at the Pontificia Universidad Católica de Chile approved this study, which was conducted according to the 1975 Declaration of Helsinki. All subjects participating in this study gave informed consent.

Daily excretion of fecal BA was measured in seven GS female patients (3 with gallstones and 4 cholecystectomized), and in 10 GS-free female subjects. All participants worked at our institution as nurse assistants. Gallstone disease was defined as either asymptomatic cholelithiasis previously diagnosed by ultrasound, or antecedent of cholecystectomy performed > 2 years prior to the study. GS are mainly of the cholesterol type in the Chilean population [2] and the morphology of patients's gallstones was of the cholesterol type in this study [27]. Criteria for selection included: a) normal weight (BMI <29); b) normal serum glucose, insulin and albumin concentrations; c) no antecedent of obesity or known changes of > 5% in habitual body weight for 6 months prior to the study; d) no antecedent of inflammatory bowel disease, or diarrhea for 2 months prior to the study; d) no antecedent of treatment with drugs affecting lipid metabolism. As a stool marker of fecal flow, each subject ingested a capsule containing 100 mg Cr_2_O_3 _(Merck, Darmstadt, Germany), with the main meals, three times daily for ten days. Daily fecal specimens were obtained for the last 3 days of the study protocol, and stored at -20°C until processing. Subjects ingested two daily meals at the Pontificia Universidad Católica de Chile. Dietary energy, cholesterol, fat, carbohydrate, protein and fiber were calculated from 7-day dietary records using the software Food Processor 7.9 (ESHA Research, Oregon, USA).

The experimental protocols of the ileal and liver biopsy studies included subjects that had a BMI <29, normal serum glucose, and no antecedent of treatment with drugs affecting lipid metabolism, including thyroid hormones, or drugs for treatment of inflammatory bowel disease. The ileal biopsy study included 14 GS-free controls (8 female, 6 male), 16 GS subjects (9 with gallstones, 7 females, 2 males and 7 cholecystectomized, 5 females, 2 males), all undergoing diagnostic colonoscopy for screening purposes. Cholecystectomized patients had cholecystectomy performed at least 3 years prior to colonoscopy. For each patient, five to seven ileal biopsy specimens were obtained from approximately 10 cm proximal to the ileocecal valve in the ileal biopsy study. The experimental protocol for the liver biopsy study included 12 GS patients (9 female, 3 male), subjected to elective surgery because of previously symptomatic GS and a control group formed by 10 GS-free individuals (5 female, 5 male), operated upon because of gastrointestinal cancer localized to the affected organ. A liver biopsy was obtained at the beginning of the surgical procedure.

A specimen of both the ileal and liver biopsy studies from each individual were fixed and stained with hematoxylin-eosin for histological analysis. All non-GS patients had a normal abdominal ultrasound for assessment of GS.

### Determination of fecal BA excretion

Fecal samples from three days of collection were subjected to alkaline hydrolysis according to de Wael et al. [28]. Unconjugated BA was extracted into diethyl ether and quantitated by gas chromatography/mass spectroscopy as previously described [29,30]. Fecal chromium from the ingested chromium oxide (Cr_2_O_3_) was determined according to Calvert et al. [31]. Concentrations of chromium and acidic sterols in feces 13 were calculated per gram of fecal homogenate. The daily excretion of BA was determined by the equation:

### RNA extraction, reverse transcription and real-time PCR analysis

Total RNA was isolated from ileal biopsies by an acidic guanidinium isothiocyanate/phenol/chloroform extraction procedure, as described by Chomczynski and Sacchi [32]. Total RNA was reverse transcribed using the SuperScript First-Strand Synthesis System for RT-PCR (Invitrogen, Bios Chile, I.G.S.A., Santiago, Chile) and random hexamers. Quantitative polymerase chain reaction (PCR) was performed in a Mx3000P Real-Time PCR System (Stratagene, La Jolla, CA, USA) using Platinum Quantitative PCR SuperMix-UDG (Invitrogen, Bios Chile, I.G.S.A., Santiago, Chile), and 25 ng of reverse transcribed RNA. Specific TaqMan Gene Expression Assays (Applied Biosystems, Foster City, CA) were performed for genes that regulate BA synthesis in the liver and BA absorption in the ileum, and for eukaryotic 18S rRNA. The primers used in the study are the following references from Applied Biosystems: *Slc10A2*, Hs00166561_m1; *Fabp6*, ILBP, Hs00155029_m1; *Abcc3*, Hs 00358656_m1; *Ostα*, Hs00380895_m1; *Ostβ*, Hs00418306_m1; *Fgf19*, Hs00391591_m1; *Cyp7A1*, Hs 00167982_m1; *Cyp27A1*; Hs00168003_m1; *Nr0B2*, Hs00222677_m1; *Nr1H4*, Hs00231968_m1. Thermal cycling conditions were 95°C for 10 minutes followed by 40 cycles at 93°C for 15 seconds, and 60°C for 1 minute. Reactions were performed in duplicate. Relative quantification of gene expression was performed using the comparative threshold (Ct) method as described by Applied Biosystems (Foster City, CA, USA). Changes in mRNA expression level were calculated following normalization to 18S expression (Applied Biosystems reference: Hs99999901_s1).

### Statistics

Data are presented as mean ± standard error of the mean. Statistical analysis was carried out by Student's *t*-test. The Ct value obtained for control and GS samples was submitted to a *t*-student analysis using REST-MCS software [33]. Differences were considered significant when p < 0.05.

## List of abbreviations

BA: bile acids; BMI: body mass index; (CYP7A1): cholesterol 7α-hydroxylase; GS: gallstone disease; (RT-PCR): real-time reverse transcription-polymerase chain reaction.

## Competing interests

The authors declare that they have no competing interests.

## Authors' contributions

JH, CH, LA and CB performed the majority of experiments; SZ, DL, JFM provided analytical tools and reagents and were involved in editing the manuscript; FN designed the study, co-ordinated and provided the collection of human material, wrote the manuscript, and provided financial support for this work. All authors read and approved the final manuscript.
